# Supplementary material for: Multifaceted and evolutionarily dynamic interactions between Caenorhabditis elegans SPO-11 and its cofactors ensure proper formation of meiotic DNA double-strand breaks
Source: bioRxiv. 2026 Aug 2:2026.07.30.741518. Preprint. [Version 1] doi: 10.64898/2026.07.30.741518 (PMC13618423; doi:10.64898/2026.07.30.741518)

bioRxiv preprint doi: https://doi.org/10.64898/2026.07.30.741518; this version posted August 2, 2026. The copyright holder for this preprint (which was not certified by peer review) is the author/funder, who has granted bioRxiv a license to display the preprint in perpetuity. It is made available under a CC-BY 4.0 International license.

Figure S1

Mapping of *top6bl* presence, [YF]ISE[ST]PXK motif emergence, SPO-11C-terminal extension, and duplication of ancient Rec114 (*dsb-1*) onto the *Caenorhabditis* phylogeny

(Left) Proteome-derived species phylogenetic tree of *Caenorhabditis* worms, with branch lengths proportional to the estimated number of amino acid substitutions per site. The three species retaining *top6BL* are highlighted in lavender. (Right) List of species shown with presence or absence of the indicated protein characteristics.

Figure S2

The SPO-11C-terminal extension correlates with an ancient duplication of *Rec114(dsb-1)* in the ancestor of the Elegans and Japonica groups

(A) (Left) Gene tree of all identified *Caenorhabditis Rec114(dsb-1/2)* genes created with IQTree3 with branch lengths showing substitutions. (Right) Rec114(DSB-1/2) protein sequences of corresponding species. The [YF]ISE[ST]PXL motif is shown in red, four simple structural motifs shared

throughout the family are highlighted in yellow, and *C. elegans* DSB-1 and DSB-2 labels are highlighted in blue. Species outside the Elegans and Japonica groups do not possess DSB-2, but some of them have recent duplications of DSB-1 (denoted by half circles next

bioRxiv preprint doi: https://doi.org/10.64898/2026.07.30.741518; this version posted August 2, 2026. The copyright holder for this preprint (which was not certified by peer review) is the author/funder, who has granted bioRxiv a license to display the preprint in perpetuity. It is made available under a CC-BY 4.0 International license.

to the protein names).
(B) (Left) Gene tree of all identified *Caenorhabditis spo-11* genes created with IQTree3, as in (A). (Right) Sequences from informative MSA regions of SPO-11 with elisions indicated by orange zigzags. From left, the regions show the N-terminal RKIEFALAD motif predicted to be involved in DSB-1 binding (coordinates around 50aa in *C. elegans*, shown in blue); the catalytic tyrosine residues (at 119aa in *C. elegans,* denoted in shown), then the C-terminal extension with two elisions involving four species (indicated by dark bars) with longer sequences, omitted here for presentation. The label "recon" in all protein names indicates manual reconstruction from the genome (see Methods).

Figure S3
Controls for pulldown and Y2H assays.
(A) The negative control 10xHis-tagged HTP-3 (409-739aa fragment) was not pulled down by MBP-tagged SPO-11 N-terminal (lane 6), C-terminal (lane 7) fragments nor MBP (lane 8), indicating that 10xHis-tag itself does not interact with MBP or MBP fused SPO-11 fragments. Proteins in the input (lanes 1-5) were visualized by Coomassie Brilliant Blue staining and proteins in the eluates (lanes 6-9) were visualized by silver-staining.
(B) All Y2H results of indicated protein fragments with 10-fold to 10^4-fold serial dilutions. Colony growth was assessed on medium lacking leucine (L), tryptophan(W) and/or histidine (H), and with 2 mM 3-amino-1,2,4-trizole (3-AT).

Figure S4 Embryonic viability analysis, assessment of protein levels by Western blot and DSB level quantification by RAD-51 immunofluorescence of *spo-11* mutants as well as FLAG-tagged control strains.
(A) FLAG tagging of SPO-11 did not interfere with embryonic viability. Embryonic viability of N2 wild type and *spo-11:3x:FLAG(wt)*. Data are presented as percentages. Statistical significance was assessed using a two-tailed t-test with Welch's correction.
(B) Western blot of FLAG-tagged SPO-11 probed with an anti-FLAG antibody. FLAG-tagged SPO-11 proteins were detected in all FLAG-tagged strains (100 worms, 24hr post-L4 stage). Anti-actin immunoblotting is shown as a loading control. Values shown below the bands represent FLAG signals that have been background-corrected using the untagged wild-type control, normalized to actin, and normalized to the corresponding FLAG-tagged wild-type control (n=2).

Figure S5
Assessment of the contribution of reduced DSB-1 protein abundance to mutant phenotypes

bioRxiv preprint doi: https://doi.org/10.64898/2026.07.30.741518; this version posted August 2, 2026. The copyright holder for this preprint (which was not certified by peer review) is the author/funder, who has granted bioRxiv a license to display the preprint in perpetuity. It is made available under a CC-BY 4.0 International license.

(A) Embryonic viability of *spo-11(3A)*, *dsb-1(RD)* and *dsb-1(RD) spo-11(3A)*. Data are presented as percentages. Statistical significance was assessed using a two-tailed t-test with Welch's correction, ****$p<0.0001$.
(B) Western blot of FLAG-tagged DSB-1 probed with an anti-FLAG antibody. FLAG-tagged DSB-1 proteins were detected in *3xFLAG::dsb-1(wt)* and *3xFLAG::dsb-1(RD)*(100 worms, 24 hr post-L4 stage). Arrowheads indicate the two specific bands detected in the blot. Anti-actin immunoblotting is shown as a loading control. Values shown below the bands represent FLAG signals background-corrected using the untagged wild type control, normalized to actin, and normalized to the corresponding FLAG-tagged wild-type control (n=2).
(C) Western blot of FLAG-tagged DSB-1 probed with an anti-FLAG antibody. FLAG-tagged DSB-1 proteins were detected in *3xFLAG::dsb-1(wt)* and *3xFLAG::dsb-1(wt)/dsb-1(we11)* heterozygous(100 worms, 24 hr post-L4 stage). Arrowheads indicate the two specific bands detected in the blot. Anti-actin immunoblotting is shown as a loading control. Values shown below the bands represent FLAG signals background-corrected using the untagged wild type control, normalized to actin, and normalized to the corresponding FLAG-tagged wild type control(n=3).
(D) Representative immunofluorescence images of RAD-51 foci in mid-pachytene oocytes (zone 4) of the gonads for each genotype indicated. Scale bar, 5 µm.

Figure S6
Embryonic viability analysis, assessment of protein levels by Western blot and DSB level quantification by RAD-51 immunofluorescence of *dsb-1* mutants as well as FLAG-tagged control strains.

(A) Quantification of RAD-51 foci in the gonads in WT and *3xFLAG::dsb-1(wt)*. Data are presented as mean±SD (bar, overall mean; dot, mean of each individual gonad; The numbers of scored nuclei in zone 1-7 were as follows: for wild type (3 gonads), 126, 141, 135, 124, 120, 99, 80; for *3xFLAG::dsb-1(wt)* (4 gonads), 130, 139, 173, 192, 207, 169, 135. Statistical significance was assessed using a two-tailed t-test with Welch's correction, *$p<0.05$.
(B) Embryonic viability of *3x:FLAG::dsb-1(wt)*. Data are presented as percentages. Statistical significance was assessed using a two-tailed t-test with Welch's correction.
(C) Western blot of FLAG-tagged DSB-1 probed with an anti-FLAG antibody. FLAG-tagged DSB-1 proteins were detected in *3xFLAG::dsb-1(wt)* and *3xFLAG::dsb-1(R116A)* (100 worms, 24hr post-L4 stage). Arrowheads indicate the two specific bands detected in the blots. Anti-actin immunoblotting is shown as a loading control. Values shown below the bands represent FLAG signals background-corrected

bioRxiv preprint doi: https://doi.org/10.64898/2026.07.30.741518; this version posted August 2, 2026. The copyright holder for this preprint (which was not certified by peer review) is the author/funder, who has granted bioRxiv a license to display the preprint in perpetuity. It is made available under a CC-BY 4.0 International license.

using the untagged wild-type control, normalized to actin, and normalized to the corresponding FLAG-tagged wild-type control (n=3).
(D) Western blot of FLAG-tagged DSB-1 probed with an anti-FLAG antibody. FLAG-tagged DSB-1 proteins were detected in *3xFLAG::dsb-1(wt)* and *3xFLAG::dsb-1(wt) spo-11(ΔC39)* (100 worms, 24hr post-L4 stage). Arrowheads indicate the two specific bands detected in the blot. Anti-actin immunoblotting is shown as a loading control. Values shown below the bands represent FLAG signal quantitation: background-corrected using the untagged wild-type control, normalized to actin, and normalized to the corresponding FLAG-tagged wild-type control (n=1).
(E) Embryonic viability of self-fertilized progeny from hermaphrodites of the indicated genotypes during the indicated time intervals after the L4 larval stage. Data are presented as mean ± SD (WT: n = 5; *dsb-2(me96)*: n = 9; *spo-11(ΔC39)*: n = 10).

Supplemental Figure 1

bioRxiv preprint doi: https://doi.org/10.64898/2026.07.30.741518; this version posted August 2, 2026. The copyright holder for this preprint (which was not certified by peer review) is the author/funder, who has granted bioRxiv a license to display the preprint in perpetuity. It is made available under a CC-BY 4.0 International license.

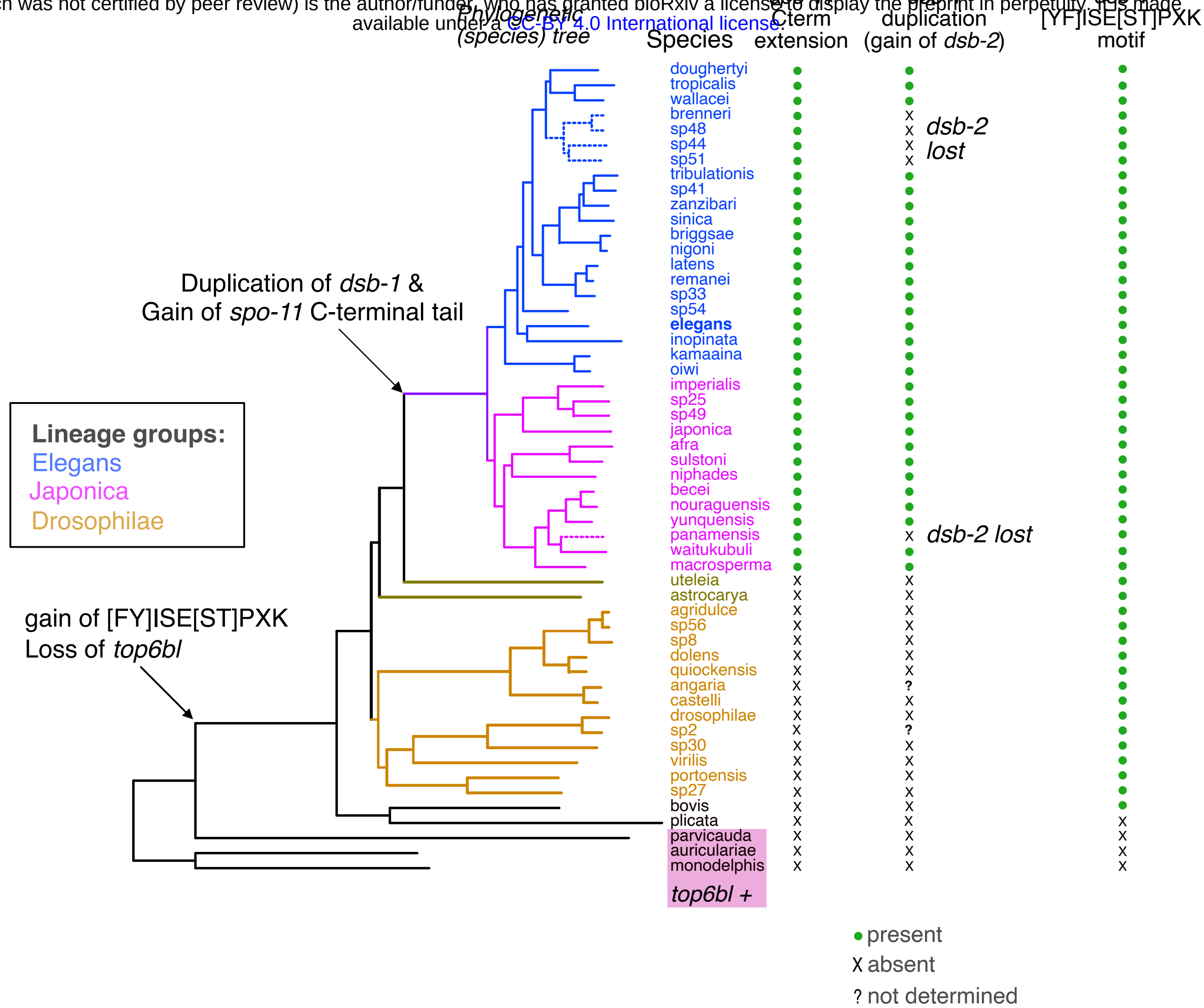

Supplemental Figure S2

A

bioRxiv preprint doi: https://doi.org/10.64898/2026.07.30.741518; this version posted August 2, 2026. The copyright holder for this preprint (which was not certified by peer review) is the author/funder, who has granted bioRxiv a license to display the preprint in perpetuity. It is made available under a CC-BY 4.0 International license.

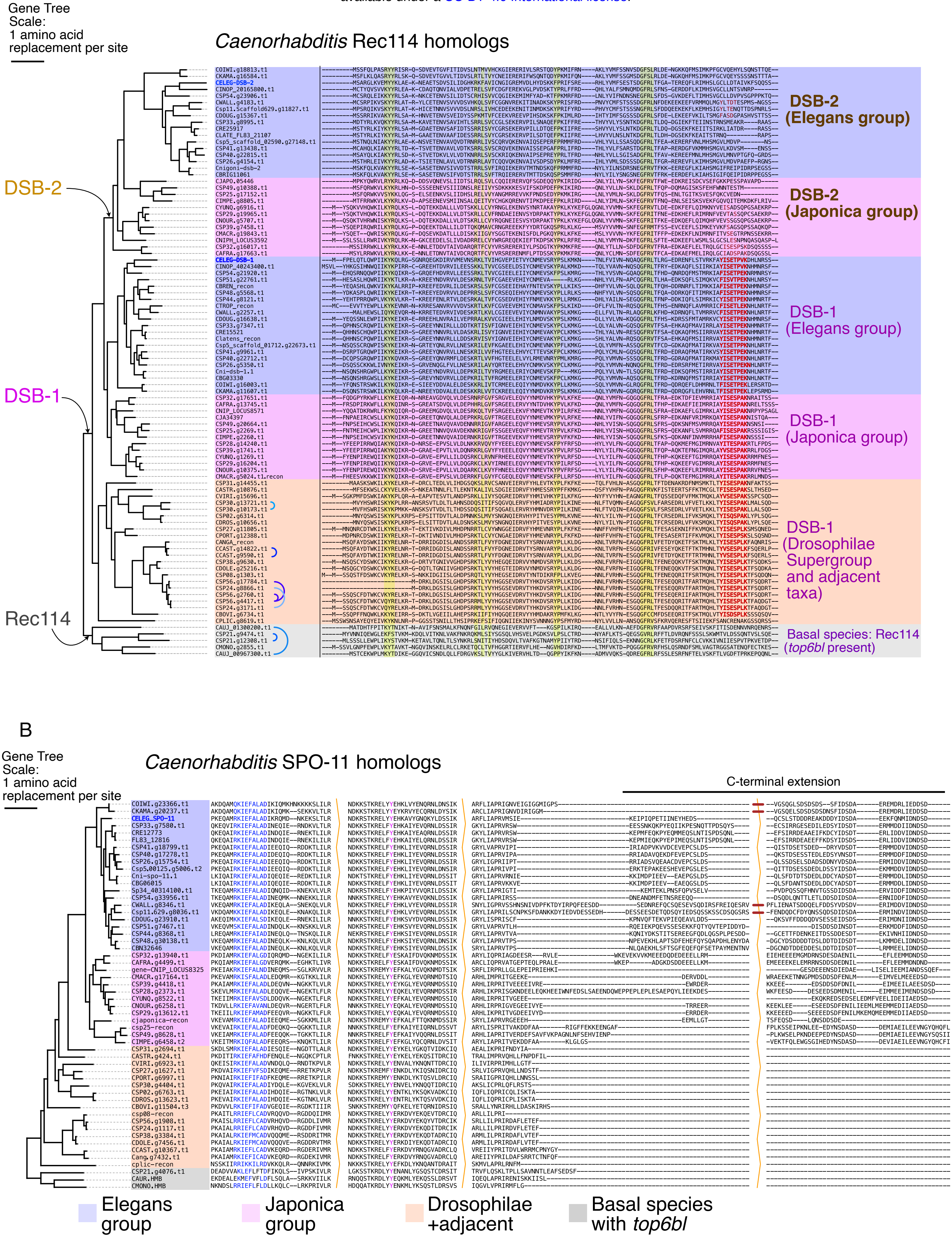

Supplemental Figure S3

bioRxiv preprint doi: https://doi.org/10.64898/2026.07.30.741518; this version posted August 2, 2026. The copyright holder for this preprint (which was not certified by peer review) is the author/funder, who has granted bioRxiv a license to display the preprint in perpetuity. It is made available under a CC-BY 4.0 International license.

A

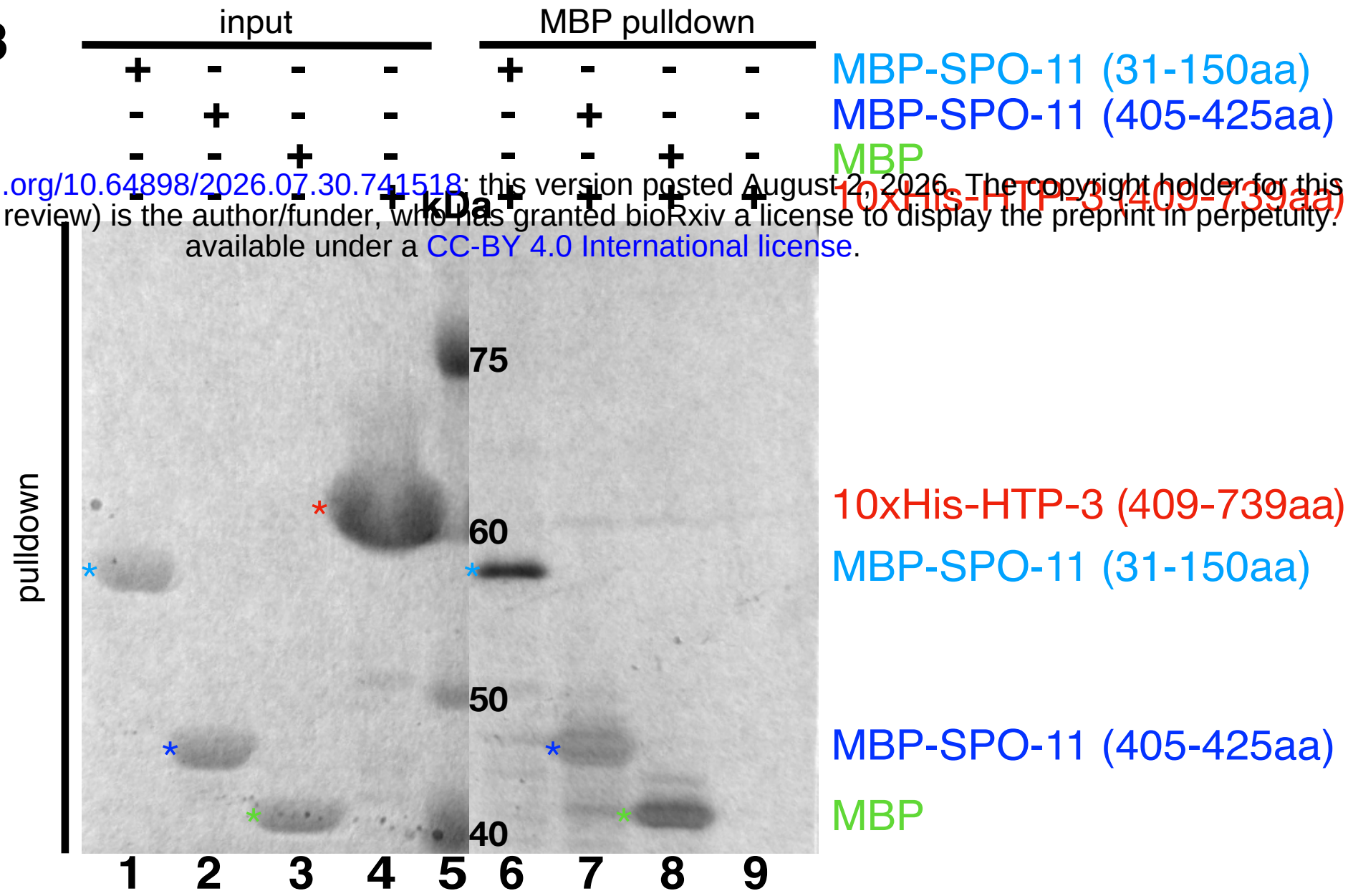

B

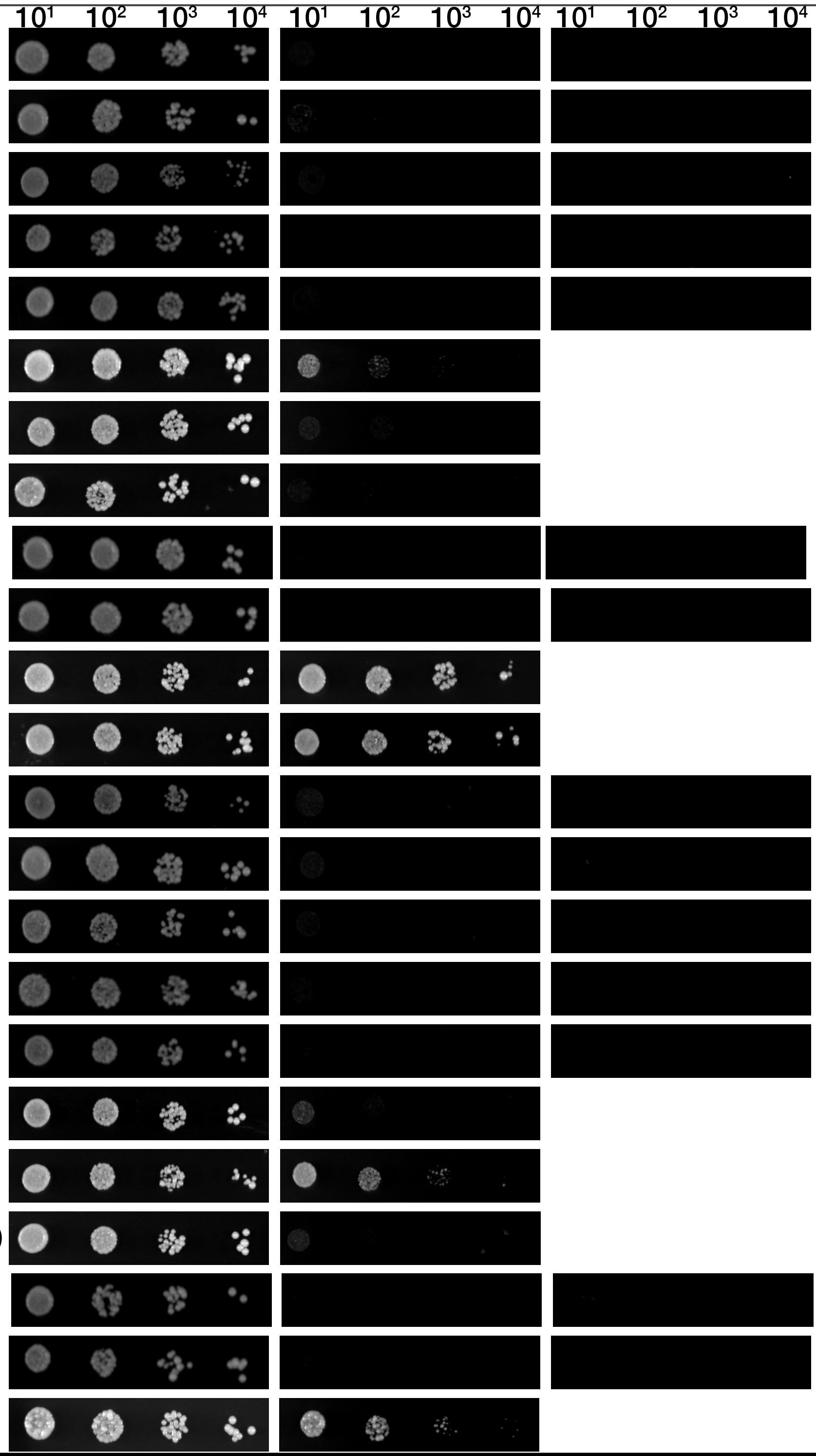

BD	AD	interaction
empty vector	empty vector	-
SPO-11(1-68)	empty vector	-
SPO-11(38-68)	empty vector	-
SPO-11(1-150)	empty vector	-
SPO-11(31-150)	empty vector	-
SPO-11(48-425)	empty vector	-
SPO-11(48-404)	empty vector	-
SPO-11(405-425)	empty vector	-
DSB-1(1-112)	empty vector	-
DSB-1(1-150)	empty vector	-
DSB-1(1-385)	empty vector	self-activation
DSB-2(1-108)	empty vector	self-activation
DSB-2(1-280)	empty vector	-
empty vector	SPO-11(1-68)	-
empty vector	SPO-11(38-68)	-
empty vector	SPO-11(1-150)	-
empty vector	SPO-11(31-150)	-
empty vector	SPO-11(48-425)	-
empty vector	SPO-11(48-404)	self-activation
empty vector	SPO-11(405-425)	-
empty vector	DSB-1(1-112)	-
empty vector	DSB-1(1-150)	-
empty vector	DSB-1(1-385)	self-activatiion

Supplemental Figure S4

bioRxiv preprint doi: https://doi.org/10.64898/2026.07.30.741518; this version posted August 2, 2026. The copyright holder for this preprint (which was not certified by peer review) is the author/funder, who has granted bioRxiv a license to display the preprint in perpetuity. It is made available under a CC-BY 4.0 International license.

A

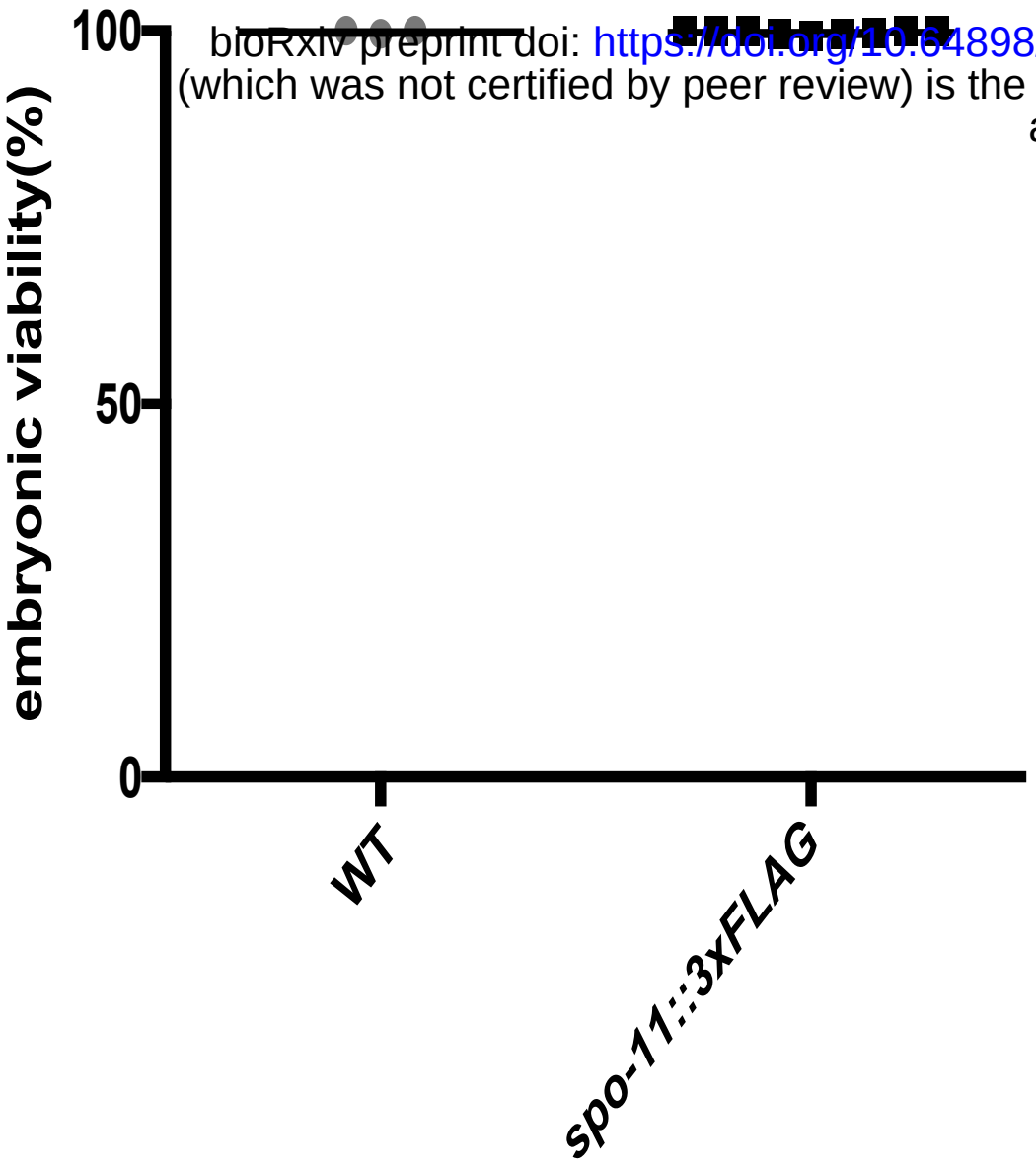

B

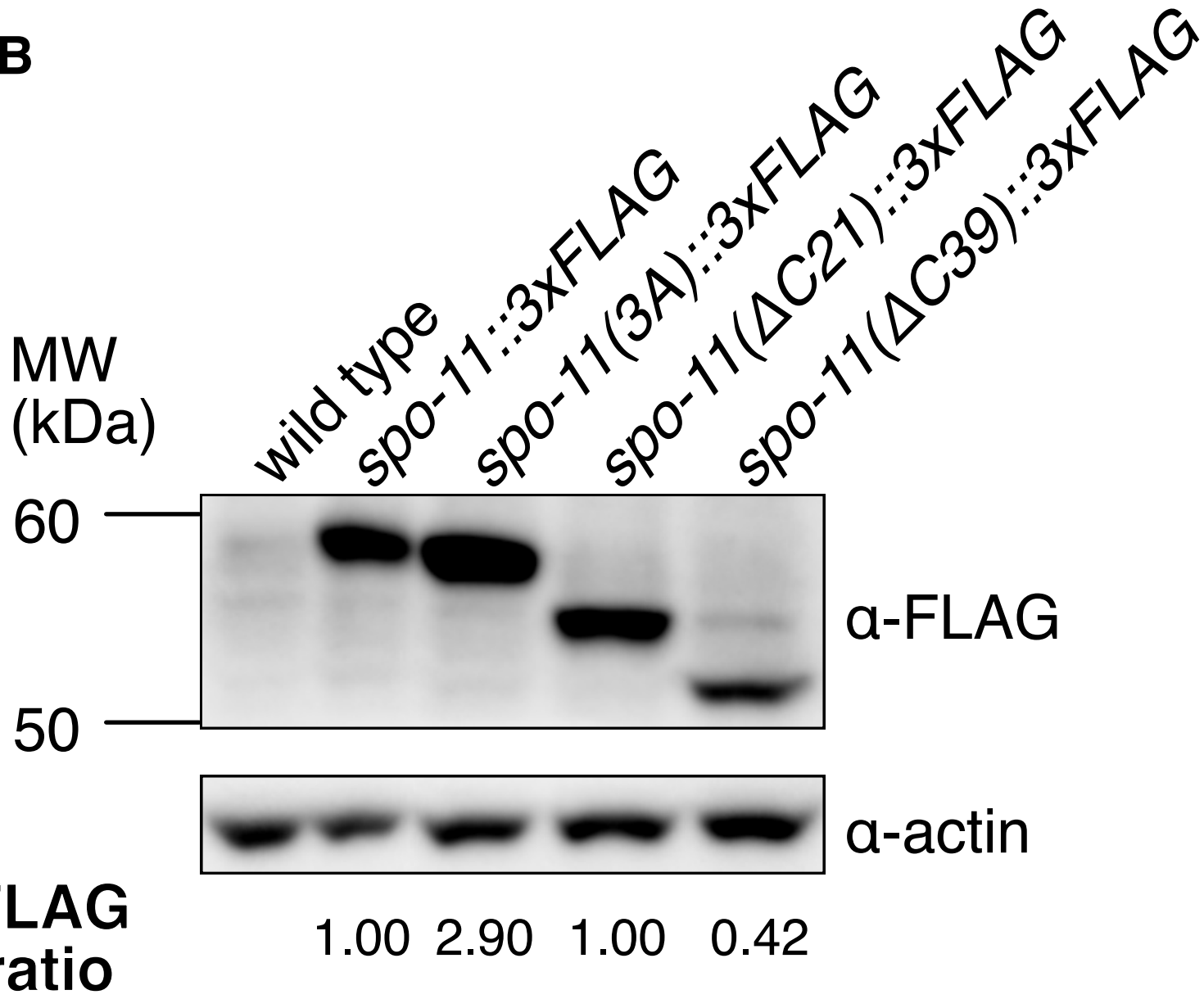

bioRxiv preprint doi: https://doi.org/10.64898/2026.07.30.741518; this version posted August 2, 2026. The copyright holder for this preprint (which was not certified by peer review) is the author/funder, who has granted bioRxiv a license to display the preprint in perpetuity. It is made available under a CC-BY 4.0 International license.

Supplemental Figure S5

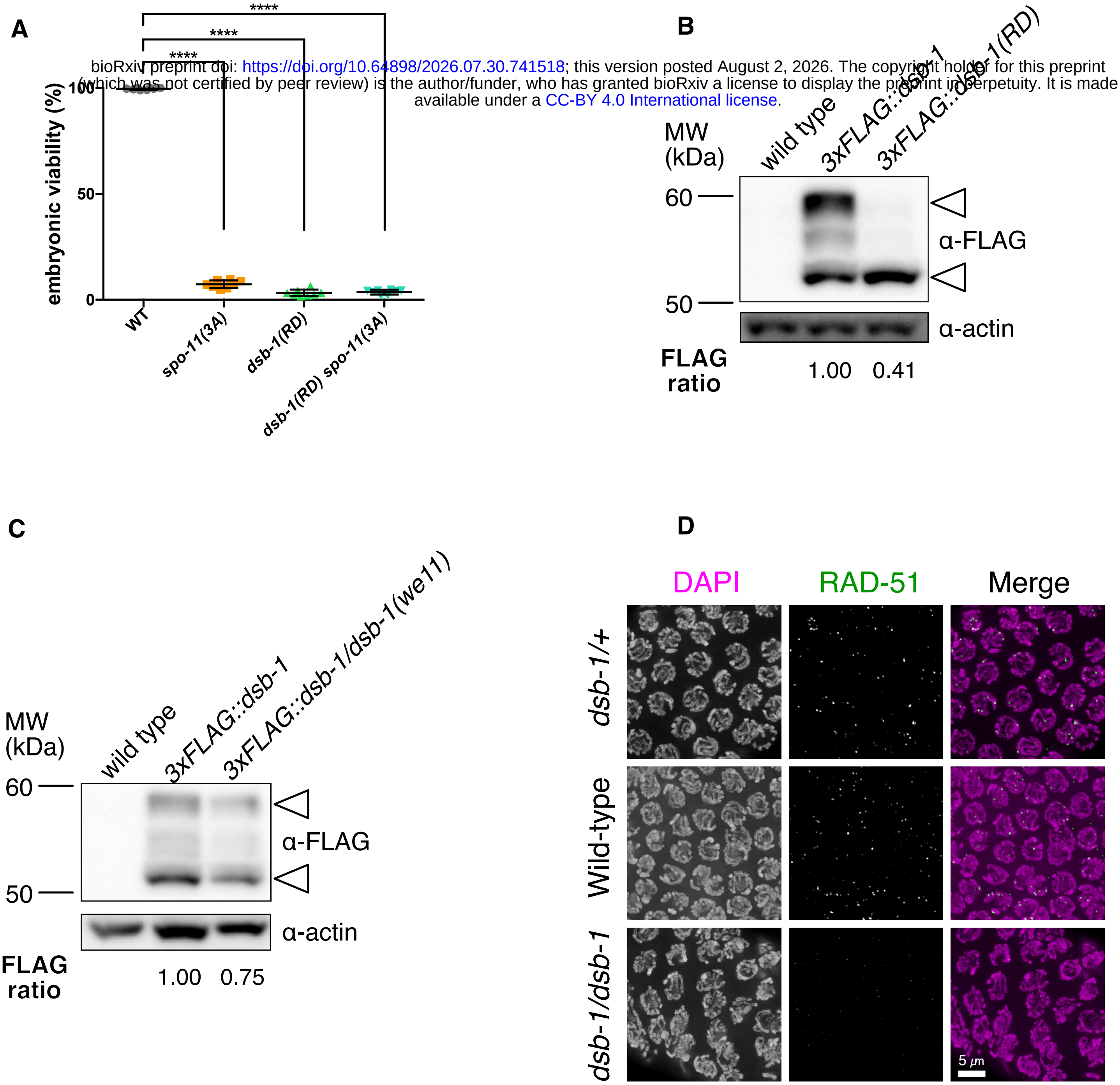

Supplemental Figure S6

bioRxiv preprint doi: https://doi.org/10.64898/2026.07.30.741518; this version posted August 2, 2026. The copyright holder for this preprint (which was not certified by peer review) is the author/funder, who has granted bioRxiv a license to display the preprint in perpetuity. It is made available under a CC-BY 4.0 International license.

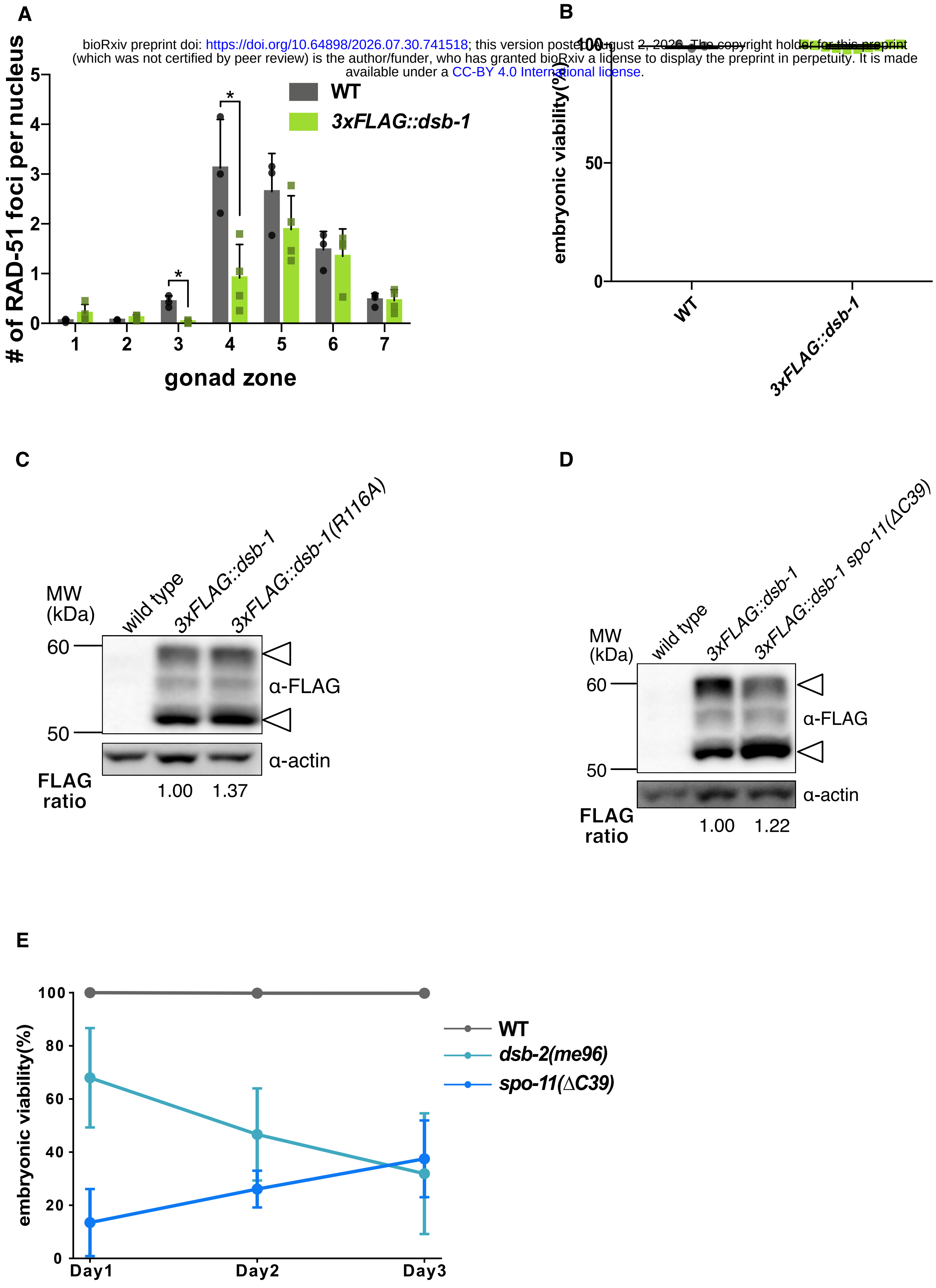

Supplement: Supplementary file 1 [file NIHPP2026.07.30.741518v1-supplement-2.pdf]
